# Supplementary figures and images for: Transcriptome Analyses Provide Novel Insights into Heat Stress Responses in Chieh-Qua (Benincasa hispida Cogn. var. Chieh-Qua How)
Source: Int J Mol Sci. 2019 Feb 18;20(4):883. doi: 10.3390/ijms20040883 (PMC6413116; doi:10.3390/ijms20040883)

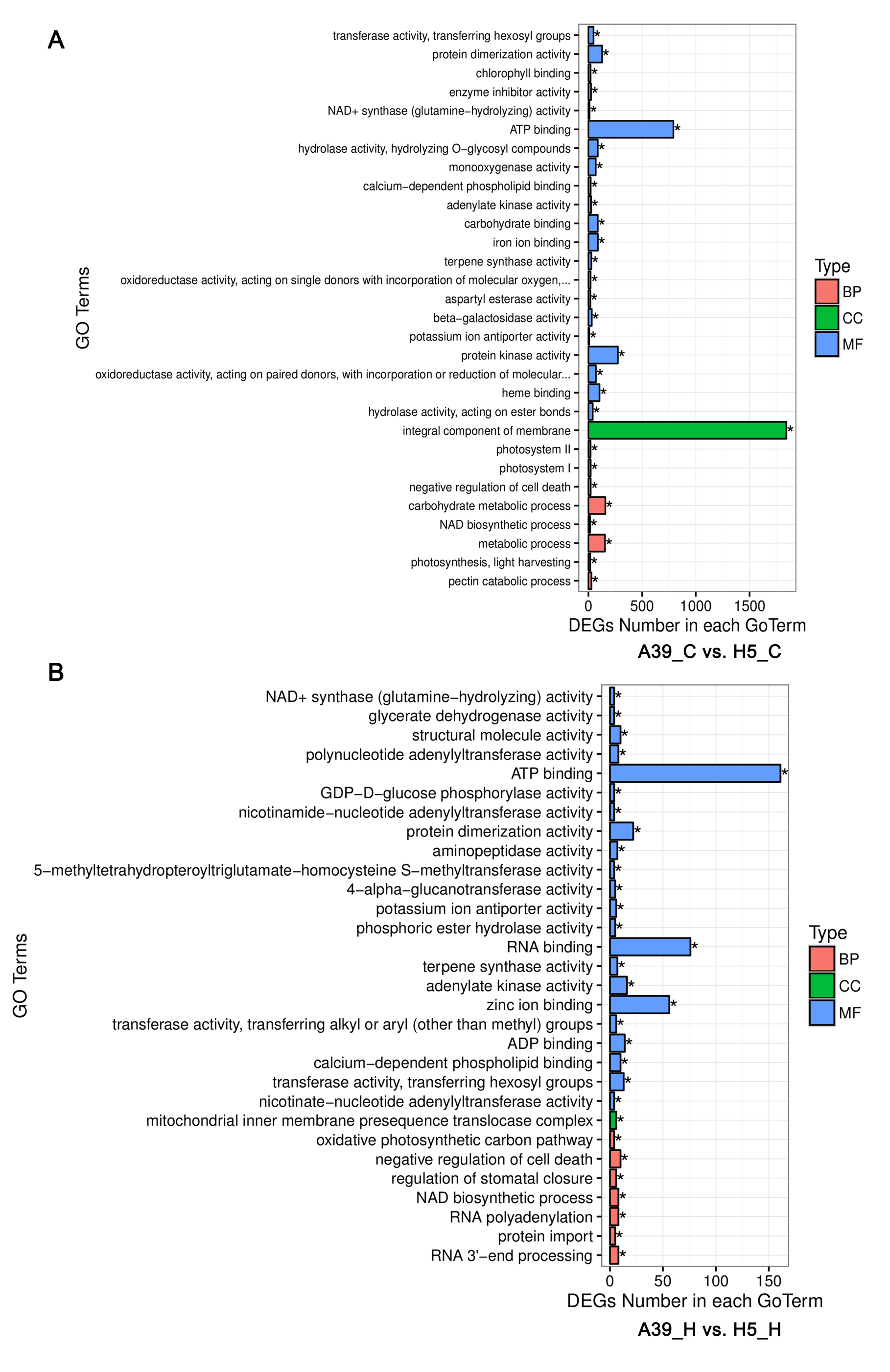

Supplement: Supplementary file 1 [file ijms-20-00883-s001.zip › Supplemental materials/Figure S1.jpg]
